# Expressiveness of an International Semantic Standard for Wound Care: Mapping a Standardized Item Set for Leg Ulcers to the Systematized Nomenclature of Medicine–Clinical Terms

**DOI:** 10.2196/31980

**Published:** 2021-10-06

**Authors:** Jens Hüsers, Mareike Przysucha, Moritz Esdar, Swen Malte John, Ursula Hertha Hübner

**Affiliations:** 1 University of Applied Sciences Osnabrück Osnabrück Germany; 2 Institute for Interdisciplinary Dermatological Prevention and Rehabilitation University of Osnabrück Osnabrück Germany

**Keywords:** wound care, chronic wound, chronic leg ulcer, SNOMED CT, health information exchange, semantic interoperability, terminology mapping

## Abstract

**Background:**

Chronic health conditions are on the rise and are putting high economic pressure on health systems, as they require well-coordinated prevention and treatment. Among chronic conditions, chronic wounds such as cardiovascular leg ulcers have a high prevalence. Their treatment is highly interdisciplinary and regularly spans multiple care settings and organizations; this places particularly high demands on interoperable information exchange that can be achieved using international semantic standards, such as Systematized Nomenclature of Medicine–Clinical Terms (SNOMED CT).

**Objective:**

This study aims to investigate the expressiveness of SNOMED CT in the domain of wound care, and thereby its clinical usefulness and the potential need for extensions.

**Methods:**

A clinically consented and profession-independent wound care item set, the German National Consensus for the Documentation of Leg Wounds (NKDUC), was mapped onto the precoordinated concepts of the international reference terminology SNOMED CT. Before the mapping took place, the NKDUC was transformed into an information model that served to systematically identify relevant items. The mapping process was carried out in accordance with the ISO/TR 12300 formalism. As a result, the reliability, equivalence, and coverage rate were determined for all NKDUC items and sections.

**Results:**

The developed information model revealed 268 items to be mapped. Conducted by 3 health care professionals, the mapping resulted in *moderate* reliability (κ=0.512). Regarding the two best equivalence categories (symmetrical equivalence of meaning), the coverage rate of SNOMED CT was 67.2% (180/268) overall and 64.3% (108/168) specifically for wounds. The sections *general medical condition* (55/66, 83%), *wound assessment* (18/24, 75%), and *wound status* (37/57, 65%)*,* showed higher coverage rates compared with the sections therapy (45/73, 62%), wound diagnostics (8/14, 57%), and patient demographics (17/34, 50%).

**Conclusions:**

The results yielded acceptable reliability values for the mapping procedure. The overall coverage rate shows that two-thirds of the items could be mapped symmetrically, which is a substantial portion of the source item set. Some wound care sections, such as *general medical conditions* and *wound assessment*, were covered better than other sections (*wound status*, *diagnostics*, and *therapy*). These deficiencies can be mitigated either by postcoordination or by the inclusion of new concepts in SNOMED CT. This study contributes to pushing interoperability in the domain of wound care, thereby responding to the high demand for information exchange in this field. Overall, this study adds another puzzle piece to the general knowledge about SNOMED CT in terms of its clinical usefulness and its need for further extensions.

## Introduction

### Background

Chronic health conditions are on the increase, constituting a long lasting disease burden for patients [1,2], and posing high economic pressure for health systems [3,4], as they require well-coordinated prevention and treatment. Among the chronic conditions, diabetes and vascular diseases causing chronic wounds are common [5]. The prevalence of chronic wounds is estimated to be 2.21 per 1000 people worldwide and is expected to increase [6]. There are different types of chronic wounds depending on the primary disease and the site, for example, diabetic foot ulcers, leg ulcers, and pressure ulcers. Among them, leg ulcers constitute the most common chronic wounds [6]. Also known as ulcus cruris or chronic leg wound, a leg ulcer is a skin defect located on the lower leg or the foot that fails to heal. Leg ulcers are caused by underlying cardiovascular diseases, most often peripheral arterial occlusive disease or venous insufficiency [7]. Furthermore, they are associated with severe complications, such as pain, immobility, local and systemic infections, and even amputations [8].

As with most chronic diseases, an interdisciplinary regimen promises to be an effective clinical therapy strategy for chronic leg wounds [9,10]. To achieve optimal treatment results for patients with wounds, physicians from multiple medical fields and disciplines such as dermatology, cardiology, surgery, primary care, specialized wound care nurses, and physical therapists are part of the interdisciplinary team [11]. In addition to the interprofessional nature of chronic wound care, it is also highly interorganizational, as patients with leg ulcers are most often treated in ambulatory settings with multiple health care providers involved, such as ambulatory nursing organizations, physician offices, and specialized wound care facilities [2].

The characteristics of chronic wound care, and its interdisciplinary and interorganizational aspects, demand information exchange about the patients between the attending medical professionals to coordinate and manage care. The healing time and disease burden can thereby be curtailed [12].

### Wound Care and Information Interoperability

To standardize and improve the documentation and communication process, various data sets for wound care were defined. Among them are the minimum data set for generic wound assessment [13], the electronic wound summary [14], and the National Consensus for the Documentation of Leg Ulcer (NKDUC) [15,16]. The minimum data set is a proposed item set for generic wound assessment in England. The electronic wound summary is a German national standard under development that describes the structure and type of information exchange at patient discharge. The NKDUC item set was published by the Institute for Health Services Research Dermatology and Nursing at the University Medical Centre Hamburg-Eppendorf, Germany. A representative consortium of clinicians, health care delivery organizations, and health insurers developed and consented this data set to standardize the assessment and record-keeping of chronic leg wounds. The data set considers international literature and medical guidelines [15,16] and is also applicable outside of Germany. The NKDUC is a comprehensive data set that goes beyond biomedical information and aims to describe the patient holistically.

Designed by the relevant professions, consented data sets such as the NKDUC are the prerequisite for meaningful record-keeping and facilitate interorganizational and interprofessional information exchange, which is a crucial process in the domain of wound care. Health information technology (HIT) systems such as electronic health records, patient health records, and patient portals can unfold the full potential of clinically consented data sets as they enable real-time, location-independent information exchange that complies with patient data protection regulations [17]. However, information sharing across HIT systems with different data models requires a standardized semantic representation of the data set’s content so that the systems can process the received clinical information appropriately [18,19]. A translation of the elements of a data set into a reference terminology guarantees semantic interoperability across HIT systems, thereby incentivizing adoption and data sharing.

Aiming for semantic interoperability in health care, the International Health Terminology Standards Development Organisation (IHTSDO) publishes and maintains the Systematized Nomenclature of Medicine–Clinical Terms (SNOMED CT). SNOMED CT promises to be the leading reference terminology for clinical terms on a global scale that enables health care professionals to exchange the semantics of data, that is, its clinical meaning [20]. SNOMED CT is a multi-hierarchical terminology that represents a clinical idea as a single SNOMED CT concept with a unique SNOMED CT identifier. Compared with other terminologies such as International Disease Classification Version 10 (ICD-10), which focuses on disease classification, SNOMED CT aims to describe the complete domain of health care entities with a high degree of specificity interrelating the concepts [21]. Hence, it promises to have the potential to cover the information elements of wound care. Furthermore, besides communication and record-keeping, standardized and interoperable clinical documentation from multiple clinical sites constitute the backbone of data-driven clinical decision support systems that may further improve treatment outcomes [18]. In recent years, an increasing number of countries have adopted SNOMED CT at the national level, including Switzerland and Austria, which joined IHTSDO in 2016 and 2018, respectively. Germany also followed suit in 2021 [22].

SNOMED CT has proven its usefulness in different areas of health care, such as, trauma information in emergency medicine records [23], cancer registries [24], and cardiology [25]. SNOMED CT therefore lends itself to be tested for chronic wounds as well to provide solutions to foster interdisciplinary leg wound care [26]. However, there is limited scientific research and evidence of how well chronic wounds can be coded in SNOMED CT; to our knowledge, there are only 2 studies. The first study mapped a set of 13 items relevant to pressure ulcers on SNOMED CT [27]. In the second study, a regional wound assessment document comprising 116 items was mapped using SNOMED CT, showing a coverage rate of 50.6% [28]. However, both studies were limited to the investigation of nursing-specific source documents. Therefore, mapping of a profession-independent, more comprehensive, and interdisciplinary consented data set to study the expressiveness of SNOMED CT promises to be rewarding in terms of revealing the potentials and limits of SNOMED CT. Such studies may also provide evidence and valuable insights for stakeholders on the use of SNOMED CT and for realizing interoperability in wound care. In addition, they might motivate clinicians to discover SNOMED CT and its usability in specific domains.

### Objective and Research Questions

The principal objective of this study is to investigate the rate at which SNOMED CT—using precoordinated concepts—covers the medically relevant expressions and terms used in the care of people with chronic wounds, particularly with chronic leg wounds. This procedure should provide evidence on the expressiveness of SNOMED CT in this medical domain and, therefore, its clinical usefulness and the potential need for extensions. Accordingly, this study pursued the following research questions:

1. Which leg ulcer concepts should be matched with SNOMED CT?
2. What is the reliability of the mapping process?
3. What is the coverage rate of SNOMED CT for leg wound terms and expressions, that is, how many source items are present in SNOMED CT?

## Methods

### Wound Care Item Set and General Methodology

To test the expressiveness and clinical usefulness of SNOMED CT in the care of patients with chronic wounds and chronic leg wounds in particular, a wound care item set based on international medical guidelines and standards with a high degree of clinical acceptance is needed. Consequently, it was decided to use the NKDUC mentioned above, which is a good example of a clinical data set to serve pars pro toto for others. The decision was made on the grounds that it is a standardized data set drawing on international recommendations and thus, features the necessary validity [15,16]. Furthermore, it embraces a rich set of terms mirroring the wound assessment, wound status, diagnostic measures, and treatments.

To meet the research objective and answer the research questions, this study comprised three main consecutive methodological blocks: First, a formal information model based on the NKDUC was designed. Second, mapping was conducted according to the Technical Report 12300:2014 of the ISO [29]. Its 21 mapping principles guided the entire mapping process (Multimedia Appendix 1). Finally, the coverage rate was determined.

### Information Model

The information model was developed using all NKDUC items of the following sections: *patient demographics*, *general medical condition*, *wound assessment*, *wound status*, *diagnostics,* and *therapy*. Therefore, only NKDUC items that were consented by the NKDUC consortium in a Delphi-based process were used [15]. Other sections were excluded, that is, *patient-related outcomes*, *patient education*, and *nutritional status*, as they exclusively referred to external sources, such as questionnaires, for example Wound-QoL (Questionnaire on quality of life with chronic wounds) [30].

Created mainly for a clinical audience, the NKDUC has a flat tabular structure. Classes and class attributes of the information model were derived from this structure, including value sets consented by the NKDUC consortium as enumerations. The information model used the class diagram notation of the Unified Modeling Language. On the basis of this model, all items (ie, class names, attributes, and value sets) constitute the set of items that were mapped to SNOMED CT.

Wherever possible, we aimed to reduce the redundant information of the NKDUC introduced through its hierarchical structure. For example, the NKDUC item *metabolic disorders* contains further detailed items such as *diabetes mellitus* and *hyperuricemia*. SNOMED CT already contains this relationship through its taxonomy, governed by the concept model [31]. In this case, we included both conditions, that is, *diabetes mellitus* and *hyperuricemia*, sparing their parent concept.

### Mapping

In the second methodological block, the NKDUC items represented in the information model were mapped onto the target terminology SNOMED CT. Mapping of the items was performed as a nonautomatic procedure. As such, it was manually conducted by 3 clinicians, that is, nurses experienced in wound care with a master’s degree in health management and a major in health informatics. Before the mapping process started, all 3 nurses were trained to work with the SNOMED CT. The training mainly focused on the logical model, which provides the fundamental structure of SNOMED CT, and the concept model, which specifies both the top-level concepts (ie, hierarchies) and the arrangement of concepts within and between these hierarchies. The mapping was conducted in 2019 using the international SNOMED CT version (January 2019) and the IHTSDO SNOMED CT browser. In this study, only precoordinated SNOMED CT concepts were used for the mapping to scrutinize the coverage rate.

Each of the 3 nurses mapped the complete NKDUC. First, they translated each source item into English. Each mapper was then instructed to use this translation to identify the semantic equivalent concept from the target terminology. As SNOMED CT concepts may share meaning but differ in granularity, the mappers were advised to scan the hierarchy of identified concepts and select a single concept that provides the highest semantic intersection between the source item and the target concept. As a result, each mapper created a simple reference set: a one-to-one relationship between the NKDUC and SNOMED CT [29].

To answer the second research question, the interrater reliability of the mapping was assessed by computing the Fleiss κ statistic. This statistic quantifies the concordance between the maps, expressing the reliability with a number ranging within the closed interval from zero to one (0≤κ≤1), with high Fleiss κ values reflecting a high agreement between the mappers [32,33]. The advantage of this statistic is that it acknowledges that agreement occurs randomly and accounts for it so that its estimate is more robust than the proportional agreement [34]. This assessment was performed for the overall mapping and the six distinct sections of the NKDUC.

### Equivalence Rating and Coverage Rate

The third methodological block included three successive steps: (1) the semantic equivalence of the previously created maps was rated, (2) the final concept was chosen, and (3) the coverage rate of the mapping was calculated (research question 3).

The equivalence rating was conducted according to the scheme described by ISO/TR 12300. According to this scheme, the semantic equivalence of the map, expressing its quality, was categorized using five degrees (Multimedia Appendix 2). The first degree describes the semantic equivalence of meaning (lexical as well as conceptual); the second degree does so too, but with synonymy. The third and fourth degrees indicate that both concepts share meaning; the former describes a broader meaning of the source concept, whereas the latter has a narrower meaning. Finally, the fifth degree indicates that mapping is impossible to achieve, as the target terminology lacks concepts that share meaning with the source concept.

The equivalence rating was independently conducted by two assessors (JH and MP), both experienced in health informatics and interoperability in wound care. Again, the reliability of the equivalence rating between both assessors was evaluated using the Fleiss κ statistic. Both assessors then selected the final concept and defined the final equivalence rating in a joint discussion. This procedure resulted in a final map for which the coverage rates were calculated.

Data were entered and stored in Microsoft Excel. Data processing and analysis were performed using the Python programming language and additional open-source packages [35,36]. The data and Python scripts are available on the web (Multimedia Appendix 2).

## Results

### Information Model

**Figure 1:**
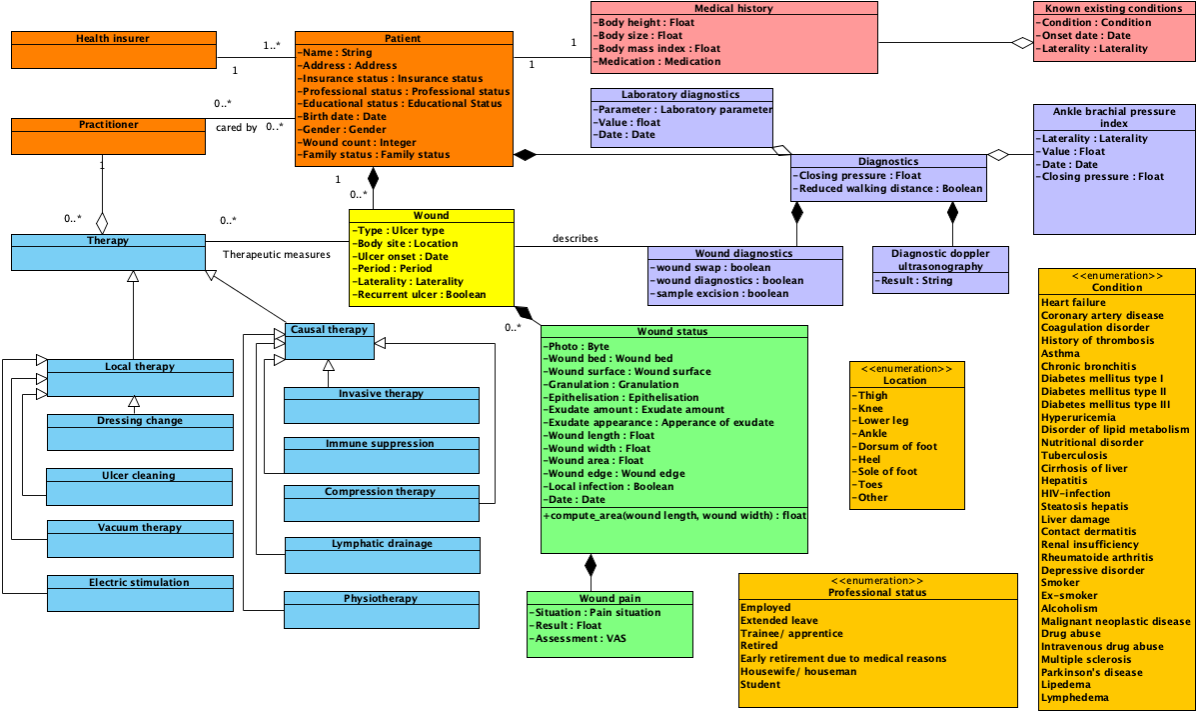
The information model (unified modelling language class diagram), with a subset of 3 selected value sets shown in dark-yellow (ulcer location, ulcer type, and condition). The class diagram only showcases the attributes for selected classes, for example, patient, wound, and wound status (orange: patient demographics, light red: general medical condition, purple: wound diagnostics, green: ulcer status, yellow: ulcer assessment, and sky-blue: therapy). VAS: visual analog scale.

The information model derived from the NKDUC revealed 268 distinct items for mapping. It included 25 classes, 66 attributes, 23 value sets, and 23 relations. Figure 1 shows an overview of the information model. The complete model is shown in Multimedia Appendix 2.

### Mapping and Reliability Rating

In the second methodological block, the previously identified 268 items were mapped first then the reliability was analyzed (Table 1). Regarding the overall reliability, the Fleiss κ value was κ=0.512. This value falls in the range of 0.41 and 0.60, which, according to Landis et al [37], can be considered a *moderate* degree of agreement. In addition, we analyzed the reliability of the mapping for each section of the NKDUC. In this context, the three mappers were most concordant for the section *general medical condition* with κ=0.754, which is considered a *substantial* agreement. The section *Patient demographics* and *Wound assessment* showed *moderate* agreement. The raters were most discordant for the sections *wound status* (κ=0.366), *therapy* (κ=0.367), and *diagnostics* (κ=0.280), which is considered as *fair* agreement [36].

**Table 1:** Interrater reliability of the three mappers that conducted the mapping (N=268).

	Reliability: Fleiss κ	Number of items, n (%)
Patient demographics	0.575	34 (12.7)
General medical condition	0.754	66 (24.6)
Wound assessment	0.568	24 (9)
Wound status	0.366	57 (21.3)
Diagnostics	0.280	14 (5.2)
Therapy	0.367	73 (27.2)
Overall	0.512	268 (100)

### Equivalence Rating and Coverage Rate

In the third methodological block, the map was finalized on the basis of equivalence rating methodology. According to the Fleiss κ value of κ=0.702, the reliability of the equivalence rating of the 2 assessors can be considered a *substantial* agreement [36]. The reliability values of the equivalence rating per section are provided in Multimedia Appendix 3.

On the basis of the equivalence rating, 79.1% (212/268) of the NKDUC items had a match in the target terminology SNOMED CT (Table 2). With respect to distinct degrees of equivalence, 43.7% (117/268) NKDUC items shared lexical and conceptual meaning (degree 1) and 23.5% (63/268) items shared meaning through synonyms (degree 2), yielding a total of 67.2% (180/268) items in the two highest categories. Furthermore, 11.9% (32/268) NKDUC items matched to SNOMED CT with semantic asymmetry combining degrees 3 and 4, among which 2.2% (6/268) items had a broader (degree 3) and 9.7% (26/268) items had a narrower (degree 4) meaning than SNOMED CT. Finally, 20.9% (56/268) items remained unmatched, described as degree 5 in the ISO/TR 12300 equivalence scheme. Table 2 breaks down the coverage rates according to equivalence categories and Table 3 shows examples for each equivalence category (see Multimedia Appendix 4 for the detailed coverage rates per equivalence category).

**Table 2:** The mapping coverage rate is presented using ISO/TR 12300 equivalence categories for the complete National Consensus for the Documentation of Leg Wounds and its sections (N=268).

Equivalence categories	Overall (n=268), n (%)	Section, n (%)					
		01: Patient demographics (n=34)	02: General medical condition (n=66)	03: Wound assessment (n=24)	04: Wound status (n=57)	05: Diagnostics (n=14)	06: Therapy (n=73)
Semantic symmetric match present (degrees 1 and 2)	180 (67.2)	17 (50)	55 (83.3)	18 (75)	37 (64.9)	8 (57.1)	45 (61.6)
Semantic asymmetry match present (degrees 3 and 4)	32 (11.9)	5 (14.7)	4 (6.1)	1 (4.2)	7 (12.3)	3 (21.4)	12 (16.4)
Semantic match absent (degree 5)	56 (20.9)	12 (35.3)	7 (10.6)	5 (20.8)	13 (22.8)	3 (21.4)	16 (21.9)

**Table 3:** Examples of the equivalence rating.

Degree of equivalence (ISO/TR 12300)	Source (NKDUC^a^)	Target	
		SNOMED CT^b^ (descriptor)	(SCTID^c^)
Equivalence of meaning; lexical, as well as conceptual	Ankle-brachial pressure index	Ankle-brachial pressure index (observable entity)	446841001
Equivalence of meaning, but with synonymy	Ulcus cruris arteriosum	Arteritic leg ulcer (disorder)	402862000
Source concept is broader and has a less specific meaning than the target concept	Skin condition	Periwound skin condition (observable entity)	700149001
Source concept is narrower and has a more specific meaning than the target concept	Wound infection	Infection status (observable entity)	405009004
No map is possible	Extent of wound area	N/A^d^	N/A

^a^NKDUC: National Consensus for the Documentation of Leg Wounds.

^b^SNOMED CT: Systematized Nomenclature of Medicine–Clinical Terms.

^c^SCTID: Systematized Nomenclature of Medicine–Clinical Terms Identifier.

^d^N/A: not applicable.

The overall coverage rate (covering degrees 1 and 2) was 67.2% (180/268). When considering the semantic equivalence for each section distinctly, the sections *general medical condition*, *wound assessment*, and *wound status* had the highest coverage rates, that is, 83% (55/66), 75% (18/24), and 65% (37/57), respectively. In contrast, the section *patient demographics* had the lowest coverage rate (17/34, 50%) and the highest number of unmatched items (12/34, 35%; Table 2). Regarding wound-specific information covered according to the two best degrees (1 and 2), *wound assessment* ranked first (18/24, 75%) and *wound diagnostics* last (8/14, 57%), with *wound status* and *therapy* ranking in between. The overall wound-specific coverage rate was 64.3% (108/168). When considering symmetric and asymmetric coverage (degrees 1-4), the wound-specific overall rate increased to 78% (131/168).

## Discussion

### Principal Findings

This study investigated the expressiveness of SNOMED CT in the domain of chronic wounds. It presents a mapping according to the ISO/TR 12300 formalism between the internationally grounded and nationally consented German data set NKDUC and the international terminology SNOMED CT. The NKDUC-based information model developed before the mapping revealed 268 items to be mapped. Conducted by 3 health care professionals, the mapping showed *moderate* reliability (κ=0.512). The coverage rate of SNOMED CT was 67.2% (180/268; symmetric match) overall and 64.3% (108/168) specifically for wounds.

### Coverage Rate

The achieved coverage rate can be regarded as satisfactory as there is a direct, symmetric match in SNOMED CT for two-thirds of all mapped items (180/268, 67.2%, degrees 1 and 2). An additional 11.9% (32/268) of the mapped items received a rating of an asymmetric match (degrees 3 and 4), which adds to 79.1% (212/268) coverage. Wound assessment ranked first (18/24, 75%) and wound diagnostics ranked last (8/14, 57%).

The mapping of a regional, nurse-specific wound care data set from the United Kingdom, Columbia, and Canada yielded a coverage rate of 50.7% [28]. In comparison, the overall coverage rates on the basis of precoordinated concepts in other clinical domains, for example emergency medicine (89%) [19], were higher but could also be as low as 30% in the case of the human phenotype ontology [38]. In this context, the coverage rates in this mapping could be deemed satisfactory.

Considering the distinct NKDUC sections, heterogeneous coverage rates became apparent. At the end of both extremes, the section *general medical condition* had the highest coverage with over 83% (55/66) and the section *patient demographics* had the lowest coverage at 50% (17/34). The former section (*general medical condition*) mainly contains a list of ulcer-relevant conditions also found in the ICD-10 classification. Past ICD-10 mappings with SNOMED CT showed that it covers those items generally well [39], which explains the high coverage rate of this section. The latter section (*patient demographics*) contains German-specific items, such as educational, marital, professional, and health insurance status, for which few matching concepts were identified in SNOMED CT. The reduced coverage rate in this section reflects the fact that the mapping was performed using the international SNOMED CT version for German-specific items. Our findings support the need to fill these gaps in the German-specific items for a national German SNOMED CT version.

Although the wound-specific sections (ie, *wound status*, *wound assessment*, *diagnostics,* and *therapy*) showed a fair to reasonable coverage rate, the mappings thereof remained incomplete. For example, 57% (8/14) of the items in the *diagnostics* sections could be mapped literally to the same term or synonym.

In addition, our investigation revealed gaps in expressing wound-care-specific terms in SNOMED CT. Clinically, interdisciplinary data sets that are based on the literature and consented by medical experts, such as the NKDUC, provide valuable insights for identifying and filling these gaps.

One approach to this is post coordination, which is used across different domains such as cardiology and clinical phenotyping data [18]. Governed by SNOMED CT’s compositional grammar and by composing existing SNOMED CT concepts, postcoordination generates semantically equivalent expressions for source terms that are unavailable as precoordinated concepts in SNOMED CT. Therefore, SNOMED CT expands its semantics and can fill gaps in a map. Postcoordination seems especially promising when the target concepts have a broader meaning (degree 4), as postcoordination can narrow down the meaning of existing concepts. Moreover, even for missing matches (degree 5), postcoordination offers a solution. For example, postcoordination would lead to the following SNOMED CT expression to code a *leg ulcer smear procedure*:

(16314007 |Microbial smear examination (procedure)|: {363700003 |Direct morphology (attribute)| = 56208002 |Ulcer (morphological abnormality)|, 363704007 |Procedure site (attribute)| = 416077002 | skin and/or subcutaneous tissue structure of lower limb (body structure)|}).

However, postcoordination is more complex and requires more effort from implementers [40], and using precoordinated concepts may facilitate the implementation. Hence, maps to precoordinated concepts promise faster adoption than their postcoordinated counterparts. Despite these disadvantages, the findings of this study reveal the need for postcoordination. Future initiatives are necessary to cover the entire domain of wound care in SNOMED CT. These initiatives require a rigorous consensus-building process to generate and validate the concepts for clinical use. In this study, our findings suggest that the wound-specific sections *diagnostics* and *wound status* may benefit the most from postcoordination as they showed the lowest coverage rates. However, when postcoordination fails, missing concepts should be added to SNOMED CT, for example, German-specific items mentioned above. Furthermore, new concepts to describe the progress of epithelization and granulation status to record wound healing are good examples to illustrate this need.

Both approaches, postcoordination and adding missing concepts, promise to close the semantic gaps identified in SNOMED CT and would allow NKDUC, and probably other documentation standards in wound care, to reach semantic interoperability. In summary, the findings show that SNOMED CT is partially ready for use in wound care documentation. However, further measures, such as those explained above, seem desirable.

### Reliability

The strength of these findings depends highly on the reliability of mapping. The overall reliability of κ=0.512 is what the reference literature describes as a *moderate* agreement between the mappers. This statistic indicates that the findings of the mapping stand on solid ground.

However, in this mapping process, rather than selecting items from a small set of options, the raters had to choose from a vast range of SNOMED CT concepts, as it provides over 350,000 precoordinated concepts. This circumstance makes it generally more difficult to find a consensus, especially for source items where similar target concepts are available. This conclusion is supported by the fact that the Fleiss κ statistic tends to decrease as the number of categories increases [41]. To increase reliability, intermediary discussions among mappers would have been beneficial. However, all mappers followed the same mapping rules to support the reliability of the mapping.

When comparing different NKDUC sections, heterogeneous reliability values could be found. For example, mappers were more discordant for concepts concerning *diagnostics* compared with those concerning *general medical conditions*. This situation may imply that sections showing low reliability are challenging to map, either because there are many similar SNOMED CT concepts with high semantic overlap, the NKDUC items are ambiguous, or both cases hold true. Reliability values and coverage rates seem to be related as lower Fleiss κ values for *wound status* (0.366), *diagnostics* (0.280), and *therapy* (0.367) tend to correspond with lower coverage rates of 65% (37/57), 57% (8/14), and 62% (45/73), respectively. Similarly, higher reliability values for *general medical condition* (0.754) and *wound assessment* (0.568) vary with higher coverage rates of 83% (55/66) and 75% (18/24), respectively. Therefore, in either case, the mapped SNOMED CT concepts in the sections with lower reliability must be validated carefully before their use in clinical practice.

### Information Model

Although the information model derived from the NKDUC primarily served as a source for identifying the items to be mapped, it also allows statements about the general validity of NKDUC by comparing this information model with others. For example, the openEHR template *wound assessment panel* and *wound presence assertions* [42], which partly represent wound phenomena, embrace similar content as the corresponding parts of the NKDUC information model. This overlap hints at the validity of this information model as well as the mapping and implications for SNOMED CT. Furthermore, it seems promising to integrate the identified SNOMED CT concepts into wound-specific openEHR archetypes and templates to enhance interoperability in the domain of chronic wound care [43].

### Limitations

There are some limitations to be considered when interpreting the results. Most importantly, as mentioned above, this study did not make use of postcoordination, which most likely limited a higher coverage rate as postcoordination usually extends the content of SNOMED CT through compositional expressions [44]. However, this study was conducted to investigate the predefined content and its coverage rate in the ulcer care domain using the NKDUC as an example of a national consented collection of ulcer-relevant items. We plan to implement postcoordination for an upcoming mapping of the NKDUC to SNOMED CT to further fill semantic gaps and improve the coverage rate required for actual implementation in systems used in clinical care. Furthermore, as NKDUC focuses on cardiovascular leg wounds, the coverage rate for items of further wound types, such as pressure ulcers, must be investigated additionally.

Another limitation of this study is the absence of a German SNOMED CT version, which necessitates an intermediary translation by each mapper, which may have introduced bias. To avoid this bias and increase interrater reliability, an additional validation step between the translation of the source concept and its mapping to SNOMED CT may have been beneficial. However, differences in the translations tend to be less biasing when synonyms are available, as is the case for SNOMED CT, and can cover these different translations. To overcome the lack of a German SNOMED CT version, a German translation group was formed by the Swiss, Austrian, and German National Release Centers in 2021 to develop guidelines and initiate translation projects [45]. The mapping that was developed in this study may support the German translation group and contribute to the translation for the domain of wound care.

### Conclusions

This study investigates the expression of SNOMED CT in the wound care domain based on a comprehensive, clinically consented data set. The results encourage the use of SNOMED CT and build the foundation for semantically interoperable systems to foster information exchange, which is crucial in the interprofessional and interorganizational setting of chronic wound care. Furthermore, the mapping followed the instructions of ISO/TR 12300 and determined the reliability as well as the equivalence and coverage rate. We thereby showcase a replicable procedure that can be used as a blueprint to produce reliable and transparent mappings in other domains as well.

Overall, this study adds another puzzle piece to the general knowledge about SNOMED CT in terms of its clinical usefulness and its need for further extensions. Semantic interoperability through SNOMED CT has become the most powerful in interdisciplinary and interprofessional scenarios across care settings, of which wound care is an excellent example.

## Appendix

Multimedia Appendix 1 The 21 mapping principles proposed by the International Organization for Standardization in its Technical Report (ISO/TR 12300) and their application in this study.

Multimedia Appendix 2 The information model, reference map and, to support reproducible research, Python script of the analysis.

Multimedia Appendix 3 Reliability of the equivalence assessment.

Multimedia Appendix 4 The coverage rate of the mapping for each equivalence category of the ISO/TR 12300 standard for each degree separately.

## Abbreviations

HIT: health information technology
ICD-10: International Disease Classification Version 10
IHTSDO: International Health Terminology Standards Development Organisation
NKDUC: National Consensus for the Documentation of Leg Wounds
SNOMED CT: Systematized Nomenclature of Medicine–Clinical Terms
Wound-QoL: Questionnaire on quality of life with chronic wounds
