# Supplementary material for: Expressiveness of an International Semantic Standard for Wound Care: Mapping a Standardized Item Set for Leg Ulcers to the Systematized Nomenclature of Medicine–Clinical Terms
Source: JMIR Med Inform. 2021 Oct 6;9(10):e31980. doi: 10.2196/31980 (PMC8529458; doi:10.2196/31980)
Supplement: Multimedia Appendix 1 [file medinform_v9i10e31980_app1.docx]

Appendix 1: The 21 mapping principles as proposed by the ISO in its Technical Report (ISO/TR 12300) and their application in this study.

| **No.** | **Principle** | **Description** |
| --- | --- | --- |
| 1 | Each map should have a (preferably single) declared purpose. | Create a one-time map of the NKDUC with SNOMED CT to primarily investigate the feasibility of SNOMED CT for the domain of wound care. |
| 2 | Scenarios are developed and articulated to define the requirements for the map table. | In this initiative, the primary focus was to obtain information about the potential coverage rate by pre-coordinated SNOMED CT concepts. |
| 3 | The map table should be in a machine-processable format. | The map is published in a csv format. |
| 4 | Identify each version of each terminological resource as a version of the map table. | The mapping is based on the information model, available at https://jnshsrs.github.io/snomed-nkduc/, which itself is derived from the tabular data provided by Herberger et al. (2017) |
| 5 | Members of the project team should have knowledge of both of the terminological resource and experience in their practical application. | Our process characterizes as a two-tier procedure. The first tier incorporates clinical knowledge from academic health care professionals (nurses) that were educated about SNOMED CT. The second tier combines clinical knowledge, terminology knowledge and knowledge about medical informatics as the team members who chose the final concepts had an education in a clinical profession (physiotherapy) and medical informatics. |
| 6 | Establish the extent to which the conventions and rules of each terminological resource will be followed. | The mapping was performed using the existing concepts in SNOMED CT. Post-coordination of concepts was not performed. |
| 7 | Custodians of terminological resources should be involved in mapping projects | Following the current mapping project, the initiative is to be continued, and further stakeholders, e.g., from medical and nursing professional associations and standardization organizations, are to be involved. |
| 8 | The automated and manual methods applied should be transparent and documented. | The mapping was conducted as a manual mapping. They are described in the corresponding scientific publication |
| 9 | Every map should describe the direction of the map. | The map is a one-directional map, where the NKDUC provides the source items are mapped with SNOMED CT concepts. The goal of this map, as described, is a one-time map whose primary purpose is the translation of the ulcer-relevant items of the NKDUC to the international terminology SNOMED CT. We did so to investigate SNOMEDCT'ss potential to cover ulcer-relevant information and its potential to encode this information. |
| 10 | The cardinality of each map should be clearly specified. | Each mapper was advised to create a one-to-one map, i.e., each source item is linked to one target concept. The results of all three mappers are then consolidated to create a final one-to-one map by two further assessors, who first conducted an equivalence rating and then chose the final item. |
| 11 | Any loss or gain of meaning should be made explicit and risk assessed. | We provide its final equivalence rating in the final map table (available as a CSV file). Based on this rating, a loss and gain of a map are described. To calculate the coverage rate, we only considered a match when asymmetry was absent. |
| 12 | All maps should demonstrate the degree of equivalence. | The degree of equivalence is provided in the final map. |
| 13 | All mapping projects should make the guidelines and heuristics applied in developing and interpreting the maps explicit when implemented. | We described the guidelines on which the mapping was based in the scientific publication. |
| 14 | Documentation supporting the map should describe the map data structures, distribution format, and licensing arrangements. | The map table and other additional files, such as the flat table of the NKDUC (the model and collection of the source items), are available in the online appendix. The required information about the files can be found there, too. |
| 15 | Every mapping project should have a quality assurance plan which includes testing and validation. | Our mapping project focused on research questions and less on the implementation of the created objects. Nevertheless, the project will be continued in terms of implementation. |
| 16 | Every mapping project should have a consensus management process. | We established a two-tier process to ensure consensus and quality of the map. The final decision was made in a joint discussion, as described in the publication. |
| 17 | Maps should be maintained and routinely updated during their lifespan. | As described, this project focused on research rather than actual implementation. However, we will use the current map based on pre-coordinated SNOMED CT concepts, and we plan to extend the mapping by using post-coordination. We plan to update this first version of our map. |
| 18 | Every map should have a maintenance and evaluation plan, which includes the mechanisms for version control. | The plain CSV file is tracked through git to monitor changes explicitly. |
| 19 | Maps should have continuous improvement processes. | We will maintain and extend the map. As described, next, we plan to incorporate the method of post-coordination. |
| 20 | Every map should have supporting documentation to assist implementation and use. | This map had a research focus; however, the complete map is available online. Further initiatives can use this map as a starting point to update and extend this current map by filling current gaps. As we did not aim for implementation, we do not provide any guidance for implementing the map. |
| 21 | Map development and maintenance is best managed through a team. | The Research Group Health Informatics conducts the maintenance of the map at the University of Applied Sciences Osnabrück |
