# Supplementary material for: Expressiveness of an International Semantic Standard for Wound Care: Mapping a Standardized Item Set for Leg Ulcers to the Systematized Nomenclature of Medicine–Clinical Terms
Source: JMIR Med Inform. 2021 Oct 6;9(10):e31980. doi: 10.2196/31980 (PMC8529458; doi:10.2196/31980)
Supplement: Multimedia Appendix 2 [file medinform_v9i10e31980_app2.docx]

Appendix 2

The information model, reference map and, to support reproducible research, Python script of the analysis is available at https://jnshsrs.github.io/snomed-nkduc/.https://jnshsrs.github.io/snomed-nkduc/
