# Supplementary material for: Expressiveness of an International Semantic Standard for Wound Care: Mapping a Standardized Item Set for Leg Ulcers to the Systematized Nomenclature of Medicine–Clinical Terms
Source: JMIR Med Inform. 2021 Oct 6;9(10):e31980. doi: 10.2196/31980 (PMC8529458; doi:10.2196/31980)
Supplement: Multimedia Appendix 3 [file medinform_v9i10e31980_app3.docx]

Appendix 3 Reliability of the equivalence assessment.

|  | **Reliability** | | **Number of Items** |
| --- | --- | --- | --- |
|  |  | **Fleiss’-Kappa** |  |
| By Section | | | |
| 01 Patient demographics |  | 0.772 | 34 |
| 02 General medical condition |  | 0.835 | 66 |
| 03 Wound assessment |  | 0.583 | 24 |
| 04 Wound status |  | 0.641 | 57 |
| 05 Diagnostics |  | 0.408 | 14 |
| 06 Therapy |  | 0.667 | 73 |
| Overall |  | 0.702 | 268 |
