# Supplementary material for: Expressiveness of an International Semantic Standard for Wound Care: Mapping a Standardized Item Set for Leg Ulcers to the Systematized Nomenclature of Medicine–Clinical Terms
Source: JMIR Med Inform. 2021 Oct 6;9(10):e31980. doi: 10.2196/31980 (PMC8529458; doi:10.2196/31980)
Supplement: Multimedia Appendix 4 [file medinform_v9i10e31980_app4.docx]

Appendix 4 The coverage rate of the mapping for each equivalence category of the ISO/TR 12300 standard for each degree separately.

| **Equivalence Categories** | **Overall**  **(n=268)** | **Chapter** | | | | | | |
| --- | --- | --- | --- | --- | --- | --- | --- | --- |
|  |  | **01**  **Patient demographics**  **(n=34)** | **02 General medical condition**  **(n=66)** | **03 Wound assessment**  **(n=24)** | **04 Wound status**  **(n=57)** | **05 Diagnostics**  **(n=14)** | | **06 Therapy**  **(n=73)** |
| Equivalence of meaning; lexical, as well as conceptual  (Degree 1) | 43.7%  (n=117) | 23.5%  (n=8) | 59.1%  (n=39) | 50.0%  (n=12) | 43.9%  (n=25) | 35.7%  (n=5) | 38.4%  (n=28) | |
| Equivalence of meaning, but with synonymy.  (Degree 2) | 23.5%  (n=63) | 26.5%  (n=9) | 24.2%  (n=16) | 25.0%  (n=6) | 21.1%  (n=12) | 21.4%  (n=3) | 23.3%  (n=17) | |
| Source concept is broader and has a less specific meaning than the target concept  (Degree 3) | 2.2%  (n=6) | 2.9%  (n=1) | 3.0%  (n=2) | 4.2%  (n=1) | 1.8%  (n=1) | - | 1.4%  (n=1) | |
| Source concept is narrower and has a more specific meaning than the target concept  (Degree 4) | 9.7%  (n=26) | 11.8%  (n=4) | 3.0%  (n=2) | - | 10.5%  (n=6) | 21.4%  (n=3) | 15.1%  (n=11) | |
| No map is possible  (Degree 5) | 20.9%  (n=56) | 35.3%  (n=12) | 10.6%  (n=7) | 20.8%  (n=5) | 22.8%  (n=13) | 21.4%  (n=3) | 21.9%  (n=16) | |
